# Supplementary material for: Can cognitive training capitalise on near transfer effects? Limited evidence of transfer following online inhibition training in a randomised-controlled trial
Source: PLoS One. 2023 Nov 10;18(11):e0293657. doi: 10.1371/journal.pone.0293657 (PMC10637678; doi:10.1371/journal.pone.0293657)
Supplement: Attachment — Submitted filename: Rebuttal.docx [file pone.0293657.s002.docx]

Rebuttal

Response: We would like to thank the reviewers for taking the time to re-evaluate the manuscript. The additional queries have been really useful and we have made some substantial changes/additions to the paper.

Reviewer 2

Thank you for the changes to the introduction and discussion, I think they improve the paper significantly.

1. I think you should add to the limitations that it had a high drop-out rate and you need to find ways to mitigate that in future work if you want to really understand what is happening.

Response: Yes, thanks for this suggestion. Added to the discussion (see lines 385-391).

2. I agree with the first reviewer that at least a drop-out analysis should be performed. Even if you don’t have significant demographic info, you can still see if the participants that dropped out had significant differences on the variables you have measured (pre-test scores, gender, etc.).

Response: Thanks, we have added this analysis in a supplementary file and refer to it in the discussion where we discuss the dropout rate. We compared the flanker performance between those that withdrew and those that stayed in the study. There was no indication that baseline cognitive performance was different between those who dropped out and those who remained in the study. There was also no indication that one gender dropped out disproportionately.

3. I agree with the authors that basic intent to treat analyses would not help in this case, but there are versions that work with participants that drop out and do not complete post-test. See this paper for example: <https://www.ncbi.nlm.nih.gov/pmc/articles/PMC6022256/>. I think it is fine if you don’t want to do this analysis, just wanted to provide some information.

Response: Thanks this looks really useful. Hopefully the analysis we have done provides some indication that the withdrawals did not bias the sample and we will consider this method in future trials.

4. I still disagree with the framing of the near transfer finding. Interpreting post-training differences is unhelpful because what you are really interested in is change from pre- to post-test. If you have differences at pre-test then post-test differences are even less useful. Sometimes, post-test t-tests are used because if randomization works correctly you can theoretically say that only post-test differences matter as pre-test differences are controlled by randomization, but as in your case randomization often doesn’t work perfectly (although your baseline differences are non-significant). In your study you have a group*time interaction and significant improvement in the active but not control group. This is a positive finding. I agree with carefully interpreting it given the other analyses, but it’s still evidence for near transfer. This sentence: “Our initial interpretation was that the interaction effect could be due to the baseline differences in the flanker scores and therefore just an artefact” in particular suggests a misunderstanding of the goals of this sort of analysis. If you had baseline differences that were erased at post-test, this could either be regression to the mean (which you mention as an option) or it could be a real intervention effect that is being masked at post-test due to real baseline differences. I recommend against post-test t-tests as meaningful analysis in an intervention design and would remove them. Overall, I don’t think this requires a huge change in the framing (it is a very tentative positive finding given the other results), but I do find it strange that you hypothesized a positive result, got a positive result, but are framing it as a negative result (not a caveated positive result).

Response: Thanks, you make a good argument that we have been overly conservative! We have reworded some sections in the discussion (lines 330-342; 403) to frame this as a positive result with some caveats, rather than dismiss it as a negative result. We have made a small change to the abstract as well to reflect this.

5. In the retention analysis, it is common to do pre-test vs. follow-up not post-test vs. follow-up. You want to know if there are differences at follow-up compared to baseline, not if differences emerged after post-test (which is unlikely). I would recommend repeating the retention analysis using pre-test.

Response: Thank you, yes this approach makes sense. We have repeated the retention analyses comparing to baseline instead (lines 283-308). We think the other analyses still have some value so we have added them as an online supplementary file. The new analyses don’t alter any of the conclusions.
